# Noninvasive monitoring of cardiac function in a chronic ischemic heart failure model in the rat: Assessment with tissue Doppler and non-Doppler 2D strain echocardiography

**DOI:** 10.1186/1476-7120-9-15

**Published:** 2011-05-26

**Authors:** Sebastian Holinski, Fabian Knebel, Georg Heinze, Wolfgang Konertz, Gert Baumann, Adrian C Borges

**Affiliations:** 1 Department of Cardiovascular Surgery, Charité Campus Mitte, Schumannstr. 20/21, 10117 Berlin, Germany; 2 Department of Cardiology, Charité Campus Mitte, Schumannstr. 20/21, 10117 Berlin, Germany

## Abstract

**Objectives:**

Feasibility of noninvasive monitoring of cardiac function after surgically induced ischemic cardiomyopathy with tissue Doppler and non-Doppler 2D strain echocardiography in rats.

**Background:**

The optimal method for quantitative assessment of global and regional ventricular function in rats with chronic heart failure for research purposes remains unclear.

**Methods:**

20 rats underwent suture ligation of the left anterior descending coronary artery via a left thoracotomy to induce ischemic cardiomyopathy. Echocardiographic examination with estimation of left ventricular wall thickness, diameters, fractional shortening, ejection fraction, wall velocities as well as radial strain were performed before and 4 weeks after surgery.

**Results:**

Mean LVEF decreased from 70 ± 6% to 40 ± 8% (p < 0.0001) one month after the operation. LVEDD increased from 7 ± 1 mm to 9 ± 1 mm (p < 0.0001), systolic anterior velocity decreased from 0.79 ± 0.25 cm/s to 0.18 ± 0.19 cm/s (p < 0.0001). Radial 2D strain was significantly reduced after myocardial infarction of the septal (18.2 ± 6.6% vs 7.0 ± 5.9%, p < 0.001), anteroseptal (17.3 ± 5.2% vs 4.6 ± 3.0%, p < 0.0001), anterior (18.9 ± 5.9% vs 5.6 ± 2.5%, p < 0.0001), lateral (21.4 ± 4.9% vs 8.1 ± 3.5%, p < 0.0001) as well as posterior myocardial segments (19.3 ± 5.2% vs 15.4 ± 5.5%, p < 0.01). Inferior segments (19.2 ± 7.9% vs 17.8 ± 7.9%, ns) did not change at all.

**Conclusion:**

It is feasible to assess dimensions, global function, and regional contractility with echocardiography in rats suffering from chronic heart failure after myocardial infarction. Particularly regional function can be exactly evaluated if tissue Doppler and 2D strain is used.

## Background

**Figure 1:**
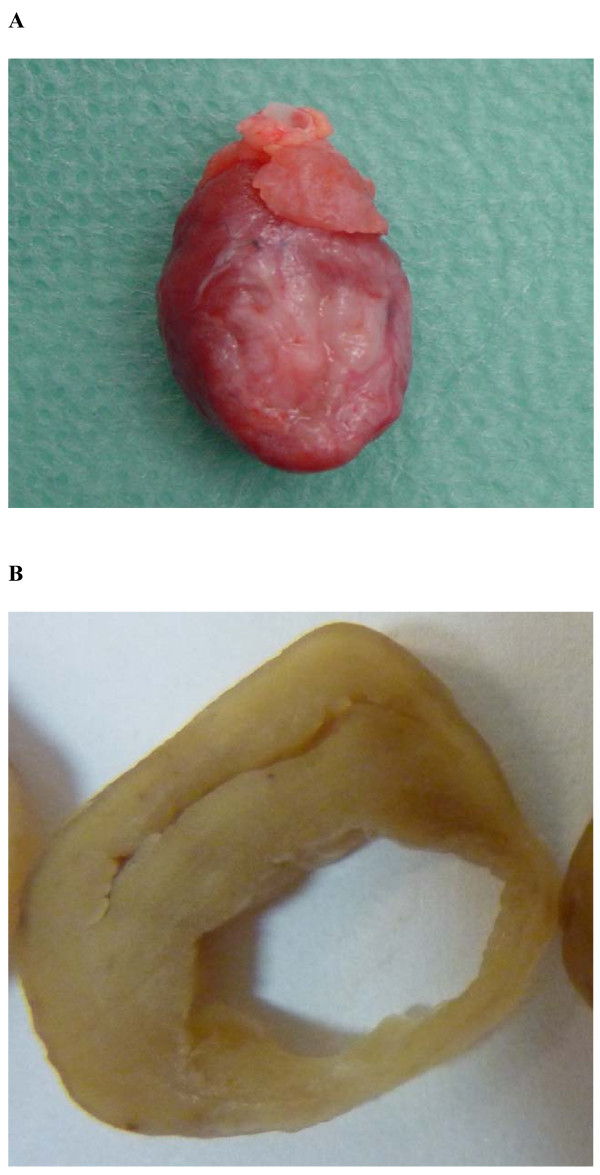
**A and B: Explanted heart with large anterior wall aneurysm (A) and cardiac slice of explanted heart one month after myocardial infarction showing semicircular left ventricular wall thinning (B)**.

Echocardiography is a well established diagnostic tool for safe and accurate assessment of cardiac function in clinical practice. Spatial resolution of latest ultrasound imaging systems approach that of magnetic resonance imaging and echocardiographic temporal resolution is better than MRI. Furthermore, new features of echocardiography namely tissue Doppler and 2D strain better detect particularly regional myocardial dysfunctions. Therefore, there is increasing interest to use echocardiography according to current standards with its newer methods also in research dealing with laboratory animals (Figure 1). However, it has been a challenge to perform reproducible echocardiography in small laboratory animals like rats. Rats are commonly used in cardiovascular and surgical models, because they are inexpensive. Moreover, microsurgery even on their hearts is well established e.g. coronary artery ligation to produce ventricular aneurysms (Figure 1). Therefore, chronic ischemic heart failure after experimental myocardial infarction in the rat is a very popular model of cardiovascular research. Recently, it has been used to study the therapeutic potential of the latest myocardial repair strategies like stem cell transplantation or tissue engineering. Accurate, serial in vivo evaluation of particularly regional cardiac function by noninvasive diagnostic measurements for this model are needed. Based on our earlier published experience of monitoring cardiac function using Tissue Doppler and non-Doppler 2D strain echocardiography of unoperated rat hearts [1] we sought to evaluate its usefulness after surgically induced ischemic cardiomyopathy.

## Methods

### Study design

A prospective, blinded animal study with paired samples was conducted in accordance with guidelines for the care and use of laboratory animals at our institutions and with approval by the local animal protection commission.

### Animal preparation and myocardial infarction

20 Lewis rats with a mean body weight of 346.6 g (median 347.5; range 315-400; SD 20.9) were obtained from a single breeding colony (Harlan Winkelmann, Borchen). Animals had free access to standard laboratory food and water ad libidum. Twelve hours of light per day were provided. All rats underwent surgical ligation of the LAD to produce myocardial infarction. They were anesthesized with xylazine (3.7 mg/kg i.p.) and ketamine (66.5 mg/kg i.p). After endotracheal intubation and ventilation a left lateral thorakotomy was performed. The pericardium was opened and the left coronary artery was proximally suture ligated using 8-0 Prolene. Myocardial ischemia could always be visualized as well as akinesia. The chest was closed and air aspirated. All rats resumed spontaneous breathing after mechanical ventilation was finished. There were no operative deaths.

### Echocardiographic Examination

Echocardiographic studies were performed before and 4 weeks after myocardial infarction using a VIVID 7 dimension system (General Electric-Vingmed Ultrasound, Horton Norway). Images were obtained using a 10S transducer (5.5-12.0 MHz) with high temporal and spatial resolution. Rats underwent the same intraperitoneal anesthesia as for the infarct surgery which was maintained throughout the echocardiographic examination. The transducer was placed directly on the shaved chest wall. A complete 2-dimensional, M-mode (according to standards of the American Society of Echocardiography), color and tissue Doppler echocardiogram was performed. Color Doppler myocardial velocity data were acquired at a frame rate of 205-230 frames/s, a sector angle of 30 degrees and an adjusted image depth of approximately 15 mm using a zoomed image window. The tissue Doppler sample volumes were tracked manually throughout the cardiac cycle, in order to measure mid-myocardial signals.

Data of 3 consecutive heart cycles were recorded digitally and analysed. Radial 2D strain was calculated from the parasternal short axis view. Strain profiles were analysed on the septal, anteroseptal, anterior, lateral, inferior and posterior myocardial segments. Peak systolic strain values were measured in each of 3 heart cycles. Systolic 2D strain measurements are based on real-time tracking of natural acoustic marker during two consecutive frames by 2-dimensional strain software (Echo Pac, GE Medical Systems) as previously described [2].

### Statistics

Statistical analysis was done using Prism^® ^5 (GraphPad Inc., La Jolla, USA). Values are expressed as mean ± standard deviation (SD) unless indicated otherwise. Paired samples of metric data were compared by t-test or Wilcoxon-test depending on the presence of Gaussian distribution. The level of significance was α = 0.05.

## Results

### Feasibility

Echocardiographic measurements were possible in all rats before and after surgery. All animals survived and we did not encounter any hemodynamic or respiratory instability during sedation and examination.

### Echocardiographic effect of LAD ligation

The echocardiographic measurements before as well as one month after LAD ligation are summarized in Table 1.

**Table 1:** Echocardiographic data of study animals before and after myocardial infarction

Criteria	Before MI (mean/SD/min/max)	1 months after MI (mean/SD/min/max)	p-value
LVEF - %	70/6/57/82	40/8/25/50	<0.0001

FS - %	35/5/26/45	17/4/10/22	<0.0001

IVSd - cm	0.2/0.1/0.1/0.2	0.1/0/0.1/0.2	NS

IVSs - cm	0.23/0/0.2/0.3	0.16/0.1/0.1/0.3	<0.01

LVIDd - cm	0.7/0.1/0.5/0.8	0.9/0.1/0.7/1.0	<0.0001

LVIDs - cm	0.4/0.1/0.3/0.5	0.7/0.1/0.6/0.9	<0.0001

LVPWd - cm	0.2/0/0.2/0.3	0.2/0/0.1/0.3	NS

LVPWs - cm	0.2/0/0.2/0.3	0.3/0/0.2/0.3	NS

V ant. sys. - cm/s	0.79/0.25/0.42/1.27	0.18/0.19/0.04/0.74	<0.0001

V post. sys. - cm/s	2.38/0.43/1.53/3.20	1.76/0.44/0.83/2.50	<0.001

2-D-strain ant.sept.- %	17.3/5.2/10.1/26.4	4.6/3.0/1.0/12.1	<0.0001

2-D-strain ant. - %	18.9/5.9/8.5/31.1	5.6/2.5/0.3/9.0	<0.0001

2-D-strain lat. - %	21.4/4.9/11.4/29.7	8.1/3.5/2.3/13.6	<0.0001

2-D-strain post. - %	19.3/5.2/8.7/31.2	15.4/5.5/7.0/24.3	<0,01

2-D-strain inf. - %	19.2/7.9/4.7/36.3	17.8/7.9/5.2/38.0	NS

2-D-strain sept. - %	18.2/6.6/7.2/33.5	7.0/5.9/0.6/28.3	<0,001

**Figure 2:**
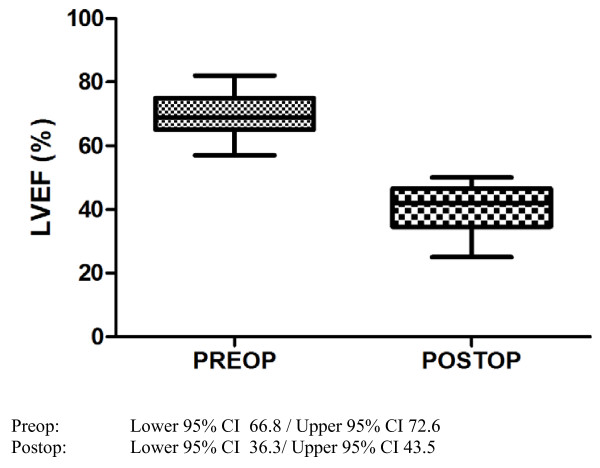
**Boxplot (Tukey) analysis of LVEF before and after myocardial infarction**.

**Figure 3:**
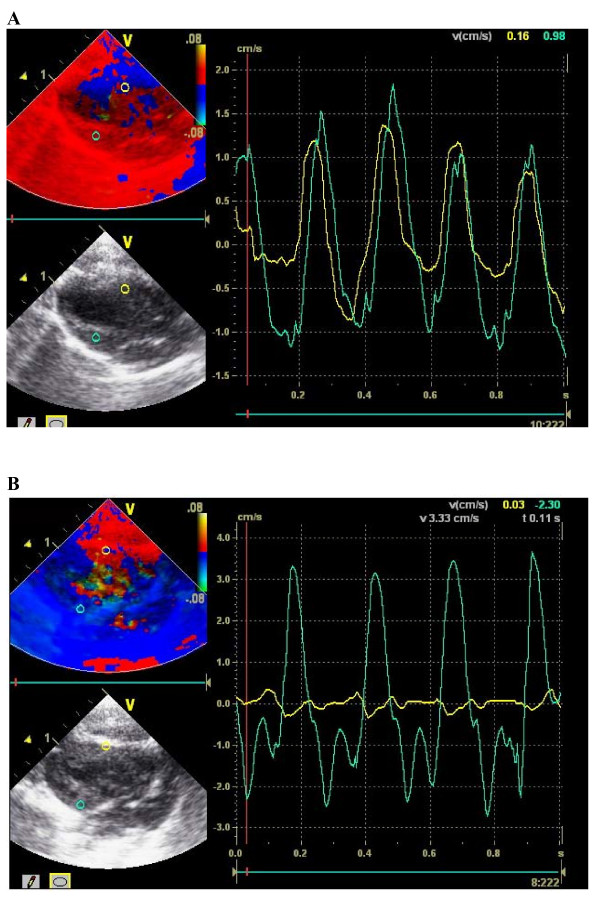
**A and B: Graphic shows systolic movement of anteroseptal left ventricular wall (green curve) and posterior ventricular wall (yellow curve) by tissue doppler before (A) and one month after myocardial infarction (B)**. See "flat" postoperative anteroseptal signal.

**Figure 4:**
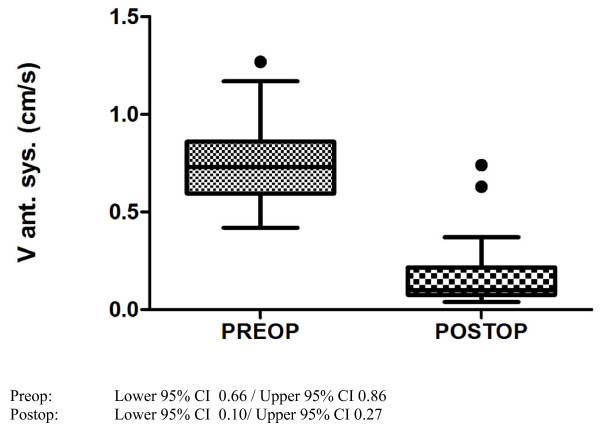
**Boxplot (Tukey) analysis of tissue Doppler measurements of systolic velocity of the anterior segment before and after myocardial infarction**.

**Figure 5:**
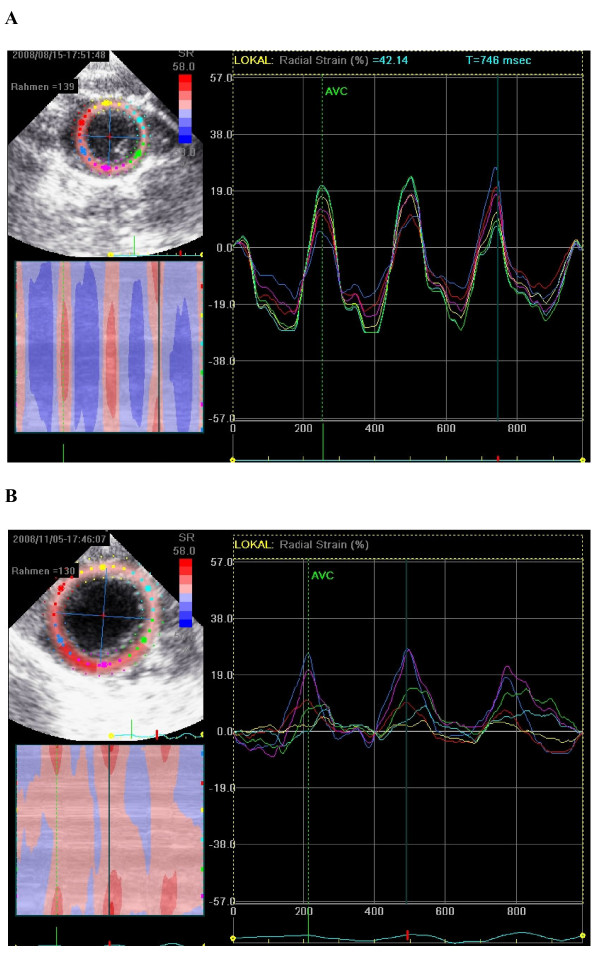
**A and B: Non-Doppler radial 2D strain before (A) and after myocardial infarction (B)**. See flattened postoperative curves particularly septal (red), anteroseptal (yellow) and anterior (light blue).

**Figure 6:**
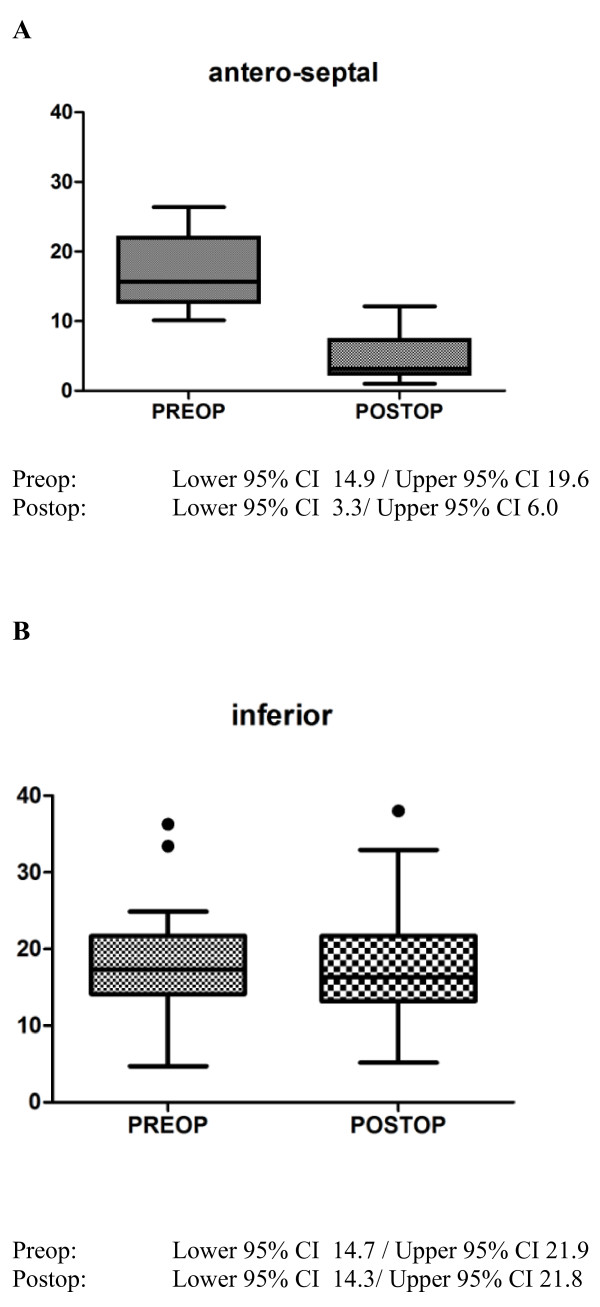
**A and B: Boxplot (Tukey) analysis of non-Doppler radial 2D strain (%) of anteroseptal (A) and inferior (B) segments before and after myocardial infarction**.

Majority of the echocardiographic mean values showed a significant change after myocardial infarction. Most of all there was a highly significant (p < 0.0001) decrease of LVEF (Figure 2), systolic anterior velocity (Figure 3 and 4) as well as an increase of LVEDD. 2-D-strain decreased particularly in the anteroseptal, anterior and lateral areas but not at all in the inferior segment (Figure 5 and 6).

## Discussion

Murine experimental myocardial infarction models are of great value to study new heart failure therapy strategies like recently the concept of cell transplantation. They are easy to perform and cost effective. However, exact monitoring of cardiac function has always been challenging due to the small size of the hearts. Invasive techniques like manometry using microcatheters had been applied but are associated with a relevant morbidity and mortality. Furthermore, they allow mainly single evaluation but serial follow ups are needed.

Transthoracic echocardiography which is a well established diagnostic tool in clinical practice was applied one decade ago to evaluate morphology as well as global ventricular function in rats despite resolution problems [3]. Left ventricular ejection fraction was the main parameter to describe global heart function. However, new features of echocardiography namely tissue Doppler and 2D strain provide precise information especially of regional myocardial function. This is particularly useful to evaluate cardiac diseases like myocardial infarction that have regional causes e.g. the occlusion of the LAD. Moreover the functional efficiency of regional therapeutic interventions like intramyocardial stem cell injection could be exactly monitored.

Based on our earlier published experience of monitoring cardiac function using Tissue Doppler and non-Doppler 2D strain echocardiography of non-operated rat hearts^1 ^we used it successfully especially to monitor regional functional changes after permanent LAD occlusion in rats. To our knowledge this is the first study demonstrating the feasibility of 2D strain echocardiography in rats for noninvasive quantification of regional ventricular function in a chronic ischemic rat model. Radial 2D strain was chosen because a good short axis view was always easy to find. Moreover it seems to be better validated than longitudinal or circumferential 2D strains. As the heart rate in rats is as high as 300/min, the frame rate had to be set between 180 and 230 for tissue Doppler measurements and between 60 and 80 for non-Doppler 2D strain, in order to obtain optimal temporal and spatial resolution.

Apparently, LAD-ligation led to a profound decrease of regional contractility not only of the anterior, anteroseptal and septal segments. Even adjacent regions like lateral and posterior segments were affected. This can be explained by the different coronary artery pattern of the rat compared to humans. In the rat the LAD is predominant and there is no true circumflex artery [4]. Therefore LAD occlusion results in a large left ventricular perfusion defect also of the lateral and posterior areas. Moreover, impairment of regional myocardial function not strictly limited to the infarct related artery territory was previously described [5].

### Limitations

A parallel group of sham operated rats could have been used to assess the existence and relevance of confounding effects not related to LAD ligation such as anesthesia.

## Conclusion

In conclusion, transthoracic echocardiography using tissue Doppler and 2D strain in rats with chronic ischemic heart failure is not only feasible. In fact it is a new powerful method for the evaluation and quantification of particularly regional cardiac function after permanent coronary occlusion in the rat. It should be used routinely and become a standard research tool for this model.

## Competing interests

The authors declare that they have no competing interests.

## Authors' contributions

SH, FK, GB and WK have designed and coordinated the study. SH and FK have equally contributed to this study and written the manuscript. SH and GH have performed the surgery and the statistical analysis. FK and ACB have performed echocardiographic measurements. All authors have read and approved the final manuscript.
